# Spatial and social disparities in the decline of activities during the COVID-19 lockdown in Greater London

**DOI:** 10.1177/00420980211040409

**Published:** 2021-08-31

**Authors:** Terje Trasberg, James Cheshire

**Affiliations:** University College London, UK; University College London, UK

**Keywords:** COVID-19, location data, regression analysis, smartphone applications, socioeconomic inequalities

## Abstract

We use data on human mobility obtained from mobile applications to explore the activity patterns in the neighbourhoods of Greater London as they emerged from the first wave of COVID-19 lockdown restrictions during summer 2020 and analyse how the lockdown guidelines have exposed the socio-spatial fragmentation between urban communities. The location data are spatially aggregated to 1 km^2^ grids and cross-checked against publicly available mobility metrics (e.g. Google COVID-19 Community Report, Apple Mobility Trends Report). They are then linked to geodemographic classifications to compare the average decline of activities in the areas with different sociodemographic characteristics. We found that the activities in the deprived areas dominated by minority groups declined less compared to the Greater London average, leaving those communities more exposed to the virus. Meanwhile, the activity levels declined more in affluent areas dominated by white-collar jobs. Furthermore, due to the closure of non-essential stores, activities declined more in premium shopping destinations and less in suburban high streets.

## 摘要

我们使用从移动应用程序中获得的人员流动数据，探索大伦敦各街区在2020年夏季新冠肺炎第一波封锁限制后的活动规律，并分析封锁指南如何暴露了城市社区之间的社会空间分割。位置数据在空间上汇总到以1平方公里为单位的网格上，并与公开移动性指标（如谷歌新冠肺炎社区报告、苹果移动趋势报告）进行交叉核对。然后将它们与地理人口分类联系起来，以比较具有不同社会人口特征的地区的平均活动下降。我们发现，与大伦敦的平均水平相比，少数民族占主导地位的贫困地区的活动减少得更少，使这些社区更容易感染病毒。与此同时，白领工作占主导地位的富裕地区的活动水平下降更多。此外，由于非必需品商店的关闭，高档购物目的地的活动减少较多，郊区商业街的活动减少较少。

## Introduction

In this study, we use data on human mobility derived through mobile applications to explore the activity patterns in the neighbourhoods of Greater London as they emerged from the first wave of national lockdown measures in summer 2020. The spatial and temporal granularity and the timeliness of the activity data enable detailed exploration of the mobility characteristics of a range of geodemographic groups and retail environments. Our analysis is motivated by earlier research (Baena-Díez et al., 2020; Jay et al., 2020) that poses a possible socioeconomic gradient in the ability of individuals and communities to adhere to social distancing measures, making certain groups of people more exposed to the virus. So far, empirical investigations of inequalities have been limited to studies focusing on spatial disparities in cases and fatalities (Bowyer et al., 2021; Office for National Statistics [ONS], 2020b), but studies looking at the socioeconomic aspects of human activity patterns during the lockdown are lacking.

The broader theme of this study is to demonstrate the potentialities of novel sources of data, such as the location data captured by smartphone apps, during public health crises. We link spatially aggregated mobile locations data to the geodemographic classifications, with the aim of identifying socioeconomic characteristics that could explain the differing rates of decline in neighbourhood activity volumes. It is hoped that our analysis will inform public health interventions that are sensitive to the underlying socioeconomic factors that can influence the uptake of mobility restrictions.

We should note that our review and analysis were finalised in September 2020 and therefore will not reflect later developments in what is a rapidly evolving situation.

## Background

### The use of smartphone data in public health crises

The number of smartphone users worldwide today surpasses 3 billion and is forecast to further grow by several hundred million in the next few years (Statista, 2020). Smart devices equipped with sensors (e.g. accelerometer and compass) and other capabilities (e.g. Cellular radio, Bluetooth, Wi-Fi, GPS) have extended our abilities to gather data on highly granular human activity patterns across large areas (D’Silva et al., 2017).

Digital data sources that provide timely information about human behaviour, especially on mobility and the physical co-presence of people (Oliver et al., 2020), are of particular value in public health crises as official data and reliable forecasts are often scarce (Ienca and Vayena, 2020). Given that the COVID-19 emergency is occurring in a digitised and connected world (Ienca and Vayena, 2020), timely data to measure changes in population behaviour (Connolly et al., 2021; Quealy, 2020) are available at large scales.

Mobile location data have been utilised to monitor the compliance of the social distancing measures put in effect to combat the pandemic (Oliver et al., 2020). The longitudinal nature of these data has enabled a baseline to be established for pre-COVID times, allowing not only the changes in mobility to be quantified (Jeffrey et al., 2020; Pepe et al., 2020) but also the recovery process of society after the crisis to be better understood (Willberg et al., 2021). Furthermore, the spatial granularity of the mobile data allows an in-depth understanding of the spatial disparities in human activity patterns during crises.

In this study, using mobile location data captured by smartphone apps, we consider the role of socioeconomic markers in explaining areal variability in the reduction of activity levels in the neighbourhoods of Greater London – the capital of the UK, which was the epicentre of the UK’s coronavirus outbreak and has been severely impacted by a high rate of COVID-19 cases and mortality.

### Smartphone location data collection

Smartphone location data are collected through software applications (‘apps’) that can be installed by the user on a smartphone and other wearable devices (Lupton, 2020). Most apps are designed to deal with a specific need (Morris and Murray, 2018) such as to help people find their destinations, provide a weather forecast, offer taxi services or monitor health and physical activity.

Location data are collected and stored through a Software Development Kit (SDK) embedded into smartphone apps. At the device level, iOS and Android operating systems combine various location data sources (e.g. GPS, Wi-Fi, beacons, network) (Pepe et al., 2020) to position the user as quickly and accurately as possible in their respective mapping and navigation products (Wang et al., 2019). The location data are further used by app developers for commercial purposes, such as location-based ad targeting, and are monetised by being sold to firms that mine the data for business insights (Romm et al., 2020). Researchers at Oxford University analysed approximately a third of the apps available in Google’s Play Store in 2017 and found that the median app could transfer data to 10 third parties (Binns et al., 2018). Although the users are given the choice to turn off the location tracking from their mobile devices (Degirmenci, 2020), the consumers do not necessarily have an indication of when their data are being collected and also have a poor understanding of how that data are used (de Montjoye et al., 2020).

### Challenges of smartphone location data

In recent years, governments have started to address the privacy concerns of in-app location data collection and sharing. For example, General Data Protection Regulation – effective in the European Union – requires the data controller (e.g. app developer) to define what is appropriate and adequate data in the context of some service delivery and to explain what happens to the personal and location data collected (Georgiadou et al., 2019).

Location privacy has received special attention since it is argued that information about an individual’s location is substantially different from other kinds of personally identifiable information (Keßler and McKenzie, 2018) because it can infer sensitive information about an individual’s social, economic or political behaviour (Georgiadou et al., 2019). For example, the *New York Times* acquired a large location dataset and was able to demonstrate that although it included no personally identifiable information it was possible to identify individuals when combined with other datasets (Thompson and Warzel, 2019; Warzel and Thompson, 2019).

Reducing the granularity of spatial or temporal information reduces the uniqueness of human mobility traces and can therefore help to mitigate these issues (Song et al., 2014). The cost to this is the introduction of further uncertainty to the data and the analytical challenges of the modifiable areal unit problem (Openshaw and Taylor, 1979). Too much spatial and temporal aggregation can also render localised patterns undetectable (González-Bailón, 2013) and limit therefore the usefulness of the data, particularly in contexts where the power of the data lies in its granularity (Scott et al., 2020).

Regardless of the challenges of maintaining user privacy, smartphone location data are similar to most consumer datasets in that they can be inherently unrepresentative of particular social groups who do not engage in the data collection process. Systematic demographic differences in smartphone ownership and proficiency (Raento et al., 2009), especially in relation to some specific segments such as the elderly (Birenboim and Shoval, 2016), may introduce generational bias (Parsons, 2020). Also, spatial disparities exist in the coverage of the data because access to mobile devices or more fundamentally the internet itself in developing countries is often limited (Parsons, 2020).

Besides limited spatial coverage, we must also be cognisant of how data were collected and, where possible, contextualise it and account for all possible fallacies that will arise from the data collection procedures (Lansley and Cheshire, 2018). Mobility data collected by smartphone applications rely on the kinds of apps that collect user location (Quealy, 2020), and the phenomena being measured by the mobile applications may be spatially dependent in some sense (Lansley and Cheshire, 2018). Understating the inherent spatial bias in the data is straightforward for the location data collected by first-party apps (e.g. Google, Apple, CityMapper), but this data are often not available for data provided by data aggregators who deliver their data through hundreds of small third-party apps.

Also, technical factors such as restricted battery life affect the reliability of smartphone-based methods (Raento et al., 2009). Consequently, quite a lot of location-based services still suffer from considerable positioning errors of GPS (usually 1–20 m in practice) (Wu et al., 2015), which limits the usefulness of the smartphone location data for analysis where the precision of the location data is essential (e.g. counting visits to certain retail stores or other facilities).

The location data in this study are spatially aggregated to 1 km^2^ grid cells so that we only know the number of unique devices per hour in each grid cell but do not have any information to construct digital traces of any of the devices. We discard observations for which the GPS accuracy is over 200 m. Also, to understand the potential bias and representativeness, we compare our dataset against other publicly available mobility metrics (e.g. Google COVID-19 Community Mobility Report, Apple Maps Mobility Trends Report) to confirm that our data show similar temporal patterns.

### Social and spatial inequalities during COVID-19 lockdown

In the United Kingdom, the attempts to slow the spread of the COVID-19 virus and to reduce the impact of acute cases on medical systems led to the implementation of unprecedented non-pharmaceutical interventions ranging from case isolation to national lockdowns (Ribeiro et al., 2020). In the absence of a vaccine or effective treatments, restricting human mobility is an effective strategy used to control disease spread (Zhou et al., 2020), but there is likely to be a social gradient in an individual’s ability to adhere to protective social distancing measures (Wright et al., 2020). A recent report published by the ONS (2020b) revealed that people living in more deprived areas have experienced COVID-19 mortality rates more than double those living in less deprived areas. Similar findings were reported by Bowyer et al. (2021), who found significant evidence of urban hotspots and a geo-social gradient associated with disease severity and prevalence in COVID-19.

Financial constraints to physical distancing may have been an important factor contributing to a higher COVID-19 burden among economically marginalised populations (Jay et al., 2020). Crowd-level data on mobile phone usage can be used as a proxy for actual population mobility patterns and provide a way of quantifying the impact of social distancing measures on changes in mobility (Jeffrey et al., 2020). Recent preliminary studies (Jay et al., 2020) have found that people in lower-income neighbourhoods have faced barriers to physical distancing, particularly the need to work outside the home. Those in elementary occupations (including cleaners, waiting staff and security guards) that tend to pay lower wages and are disproportionately held by minority populations, as well as people with lower educational attainment (Mongey and Weinberg, 2020), are much less likely to be able to work remotely than employees in higher-paying jobs (ONS, 2020a). While people with higher education and white-collar office workers were able to switch to remote working, blue-collar employees had to work on-site and risk being exposed to the virus (Dingel and Neiman, 2020).

In this study, we explore the spatial and social disparities in the decline of activities, but instead of looking at specific measures of the neighbourhood (e.g. median income, average education level, etc.) as has been done in previous studies (e.g. Jay et al., 2020), we propose using geodemographic classifications to assess the changes in the activity patterns of different urban communities. Geodemographic classifications have been created by clustering demographic data and are designed to accumulate a complex body of information about a population, making them a more robust reflection of the social, economic and demographic characteristics of a neighbourhood. A similar approach of linking location data and open geodemographics was applied in the study by Liu and Cheng (2020), who integrated smart card data with workplace classification to understand traveller behaviour, in particular the passenger composition of the stations alongside the two Night Tube routes. Geodemographic classifications have also been used in health research for targeting neighbourhoods in public health campaigns (Petersen et al., 2011) and measuring inequalities in health (Abbas et al., 2009).

## Case study

The aims of this case study are twofold. In the first part, we describe our sample mobile locations data and compare temporal patterns with other mobility metrics released by Google and Apple in their respective ‘Mobility Reports’. The availability of multiple data sources measuring similar phenomena allows verification and cross-checking of the patterns.

In the second part of the case study, we analyse the discrepancies in the decline of activity levels. We first add contextual information that indicates the social, economic and demographic characteristics of a neighbourhood to the 1 km^2^ grid cells covering Greater London and study the dependency between the population characteristics in the neighbourhood and the decline of the activity levels during the lockdown.

### Data

The anonymised smartphone location data applied in this study are provided by Huq Industries (https://huq.io/). Technical data (including location information, date and time) about a device are collected using mobile app SDK embedded within one of their mobile app partners’ apps. The data are captured only when the mobile app partner has obtained users’ prior explicit consent to SDKs collecting the data (more information: https://huq.io/privacy-policy/).

We use a subset of Huq’s database for the time period of January to July 2020, covering the Greater London region. There are in total around 500 mobile partner apps that contribute data to the database in this period; however, the majority of the apps are in use infrequently. We exclude temporary apps so that the sample used in the study includes only data collected by the apps that were in use consistently throughout the study period. So, the sample data includes mobile location information collected by 146 apps. The names of the apps are hashed and not known to the researchers.

The location data has been collected from 308,311 unique devices during the study period. However, as the (location) data are collected only when the device interacts with an app, the panel of unique devices present at each time point varies. The data are collected from ∼ 93,000 unique devices in January, but from ∼ 42,000 unique devices in April. On average, a device is active for 24 days during the study period (on average eight days per month).

In the next step, data are spatially and temporally aggregated so that no individual trajectories can be detected. First, the location information (captured as latitude and longitude) is replaced by a grid ID through spatially joining the data points to a 1 km^2^ grid. There are in total 1731 grid cells covering the Greater London Authority (GLA) area. A 1 km^2^ grid was chosen as this level of spatial aggregation preserves the spatial patterns but also allows the data to be easily linked to a geodemographic classification using population-weighted centroids (explained in section ‘Linking mobility data and geodemographic variables’).

Next, we count the number of unique devices that have been present in each grid cell per hour. We refer to the count of devices in the grid cell as the level of activities. The activity measure reveals that a device has been in a certain grid cell, but not how long it stayed there or whether it was passing through. To obtain the general daily activity measure, we add together the hourly activities for the respective date. The daily values are then converted into percentages that show the ratio between activity levels at a given date and the baseline period (3 January to 6 February) (rescaling is explained in sections ‘Addressing the representativeness of case study data’ and ‘Rescaling and aggregating data’). As the lower numbers tend to create outliers when transformed into percentages, we add a further data cleaning step where we remove the grid cells that have been visited by fewer than 10 unique devices on any day during the study period. This excludes 559 (32%) grid cells located mainly in the outskirts of Greater London (see Figure 3).

To sum up, the final sample data have the following attributes: *cell ID, date, total activities*. The data have been collected through 146 apps from 308,311 unique devices and are spatially aggregated into 11,72 1km^2^ cells covering the Greater London area.

#### Addressing the representativeness of case study data

We compare the temporal patterns in the Huq activity data to various other activity metrics made available by Google and Apple in their respective ‘Mobility Reports’. The comparison includes 10 different activity measures, each of which indicate the change in certain types of activities during the COVID-19 pandemic.

Google activity metrics show the percentage of change in the number of visits to the places of interest relative to a median value of the five weeks from 3 January to 6 February 2020 for each weekday. The places of interest are clustered into four groups: *Parks* (includes visits to local parks, national parks, etc.), *Transit Stations* (tube, bus and train stations), *Grocery & Pharmacy* (grocery markets, pharmacies, etc.) and *Retail & Recreation* (restaurants, cafes, shopping centres, etc.). The report also includes information about the change in the average stay at places of residence calculated based on the change in the average amount of time (in hours) that users spend at home (Aktay et al., 2020). A further metric is available for places of work calculated as the percentage of change in the number of unique users who spend more than one hour per day at their workplace. The aggregation and anonymisation process applied in creating the activity metrics is described in Aktay et al. (2020). The location data are derived from 1 billion monthly users globally who have turned on the ‘Location History’ in the Google account settings and allowed the Google Maps web mapping application to store the device’s location (Russell, 2019). The earliest date that this data is available is 15 February 2020 and the data are updated weekly.

Apple mobility metrics, released in April 2020, reflect the changes in the requests for directions in Apple Maps during the COVID-19 pandemic. The mobility index is calculated separately for requests made for driving, walking and transit, and the mobility index is defined as the percentage of request volume relative to the number of requests made on 13 January 2020. The report is updated daily.

Apple measures are relative to 13 January 2020, whereas Google measures are calculated against the median activity level of the five-week period of 3 January to 6 February for each weekday. Apple’s single-day baseline preserves the variations across the weekdays, whereas the longer baseline used by Google where each weekday is compared against the median value of the baseline for that weekday removes the weekly cycles in the data. As the Google Mobility Index has the most limited availability, we rescale the Huq and Apple data using the methodology proposed by Google. For the Huq data, this could be done to the same baseline period (3 January to 6 February), but Apple data were rescaled using a slightly shorter time period (13 January to 6 February) as the data are not available for earlier dates. The missing values (Apple Mobility Index has missing values for 11–12 May) are replaced by linear interpolation using *na.approximate()* function from the zoo package (Zeileis et al., 2020) in R (R Core Team, 2019).

**Figure 1.**
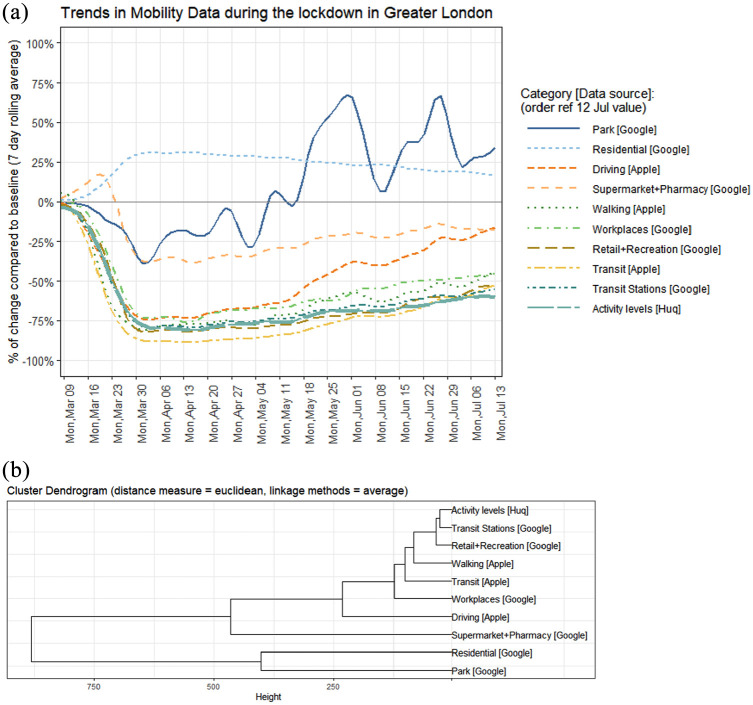
Cluster analysis of the mobility metrics. (a) Trendlines pertaining to the change in different activities. The *Residential, Park* and *Supermarket & Recreation* categories show significantly different patterns from the remaining seven activity metrics including the Huq activity data. (b) Dendrogram showing the clusters of similar activity metrics. Huq activity data are most similar to Transport Stations metrics.

After rescaling the metrics, we visualise the data (see Figure 1) and calculate the similarity, expressed as Euclidean distance, between all the time series for the period of 2 March to 13 July 2020. The similarity measures are then fed into a hierarchical clustering algorithm. Hierarchical clustering partitions data into different levels that resemble a hierarchy, which provides an easy way to inspect the similarities in the nested grouping of patterns and levels at which groupings change. Unlike other popular clustering techniques such as K-means and PAM, hierarchical clustering does not require the number of clusters to be defined in advance. We use the average-linkage algorithm that (unlike single-linkage and Wards linkage methods) is robust to outliers, which is important for our analysis as the outliers have not been removed since they carry relevant information about how the different time series react to temporary external factors (e.g. weather or public holidays).

The analysis is conducted in R using the *dist()* function from the stats package (R Core Team, 2019) to compute the distance matrix and the *hclust()* function from the cluster package (Maechler et al., 2019) to perform hierarchical cluster analysis. The factoextra package (Kassambara and Mundt, 2020) function *fviz_dend()* that draws dendrograms is used for visualising.

The trends in activity metrics and the dendrogram showing the clustering hierarchy are visualised in Figure 1. The *Residential* category, which measures the change in the duration of the time people spend at home, rises around 25% during the lockdown as people spent more time at home. However, the magnitude of the change (38% at its peak) was not as significant as in some other categories (e.g. −89% at *Transit*, −83% at *Retail & Recreation*) because people spent a lot of time at their places of residence also before the lockdown. The visits to the *Park* category also increased, although as the metrics are not seasonally adjusted the change in activities most probably reflects changes in the weather rather than the changes in mobility caused by the lockdown. The *Grocery & Pharmacy* category combines visits to the locations that are considered to be essential trips. There was a spike in activities in the *Grocery & Pharmacy* category in the week before the lockdown as people started to shop in bulk ahead of the lockdown. Also, the majority of the places included in this category stayed open throughout the lockdown. Therefore, there was less of a decline in activities in the *Grocery & Pharmacy* category compared to the other categories (e.g. *Retail & Recreation*).

The patterns in the rest of the seven categories are similar to each other. There was a decline in the week before and during the first week of the lockdown, where activities dropped up to 80% compared to the baseline. The lowest levels of activity were recorded around the Easter holidays between 10 and 13 April. The *Transit* and *Retail & Recreation* categories showed the most decline, dropping by as much as 89% and 83% respectively. There has been a steady incline in activities in all categories since the Easter holidays. The *Driving* category has recovered faster compared to the other categories which saw a similar decline (e.g. *Transit, Transit Stations*, etc.).

The hierarchical clustering results show that trends in Huq activity levels are most similar to the *Transit Station* category. Both categories reach the lowest level of activities on 13 April, where transit stations had 80% and Huq activity levels 81% fewer activities compared to the baseline. The *Retail & Recreation* and *Workplaces* categories also show similar patterns to Huq activity levels, but *Retail & Recreation* saw more decline because most of the shops were closed during the lockdown and *Workplaces* saw less decline over the weekends as those areas had low activity volumes over the weekends even before the lockdown.

### Methodology

#### Linking mobility data and geodemographic variables

We aim to obtain a more detailed view of the discrepancies between neighbourhoods within Greater London. This part of the case study includes only Huq data because Google and Apple mobility data are not available in the same level of detail. From the comparison with other available activity metrics from Google and Apple Mobility Reports, we conclude that activity levels in Huq data are representative of the mobility of the ambient population in Greater London.

We assign geodemographic categories to the 1 km^2^ grids and examine the change in activities within each geodemographic classification across Greater London. These classifications are created by clustering demographic attributes into groups that exhibit similar characteristics at a range of geographies. The classifications selected for the analysis are shown in Table 1. Our choices reflect the desire to capture residential (LOAC and IMD) characteristics as well as those in areas of employment (LWPZ), a distinction that is particularly pronounced in London. In addition, the plight of retail areas has garnered significant attention and is seen as key for managing the economic recovery, particularly in the face of successive closures and re-openings in an era of local lockdowns. We have therefore included a typology of retail centres in the analysis (Dolega et al., 2021).

**Table 1.** Detailed description of geodemographic classifications and other area characteristics included in the analysis.

Geodemographic classifications and other area characteristics	
Classification	Details
London Output Area Classification (LOAC) (Longley and Singleton, 2014)	Captures the characteristics of the residential population in Output Areas (OAs) using data from the 2011 census. OAs are compact and homogeneous areas with a target size of 125 households built from postcodes. The classification uses a combination of over 60 census variables to classify all OAs, based on their similarities, into eight Super Groups and 19 Groups.
Index of Multiple Deprivation 2019 (IMD) (McLennan et al., 2019)	The IMD is calculated for every Lower-layer Super Output Area (LSOA) in England. LSOAs are created by merging OAs and have an average of approximately 1500 residents or 650 households. The index is based on 39 separate indicators, organised across seven distinct domains of deprivation (income, employment, health, education, crime, housing and services, living environment) that are combined and weighted to calculate the IMD. In the case study, we apply deprivation deciles, where Decile 1 represents the most deprived 10% of neighbourhoods and Decile 10 represents the least deprived 10% of neighbourhoods.
London Workplace Classification (LWPZ) (Singleton et al., 2017)	Workplace Zones (WZs) have been created by splitting and merging OAs to produce a workplace geography that contains consistent numbers of workers (Martin et al., 2013). Effectively, this is a geographic redistribution of the usually resident population who are in work, allocated to their place of work. Unlike the LOAC and IMD Index which are based solely on information derived from the census data, the LWPZ uses supplementary data from other data sourced through the CDRC, the ONS and Transport for London, including variables pertaining to the dynamism and attractiveness of workplace settings, the retail structure and accessibility. A total of 92 variables were used to classify the 8154 WZs in London into five Groups and 11 Subgroups.
Retail Centres (Dolega et al., 2021)	Retail centres are defined as distinctive areas of increased concentration of retail activity. The geography of retail centre boundaries was designed by Pavlis et al. (2018) and the typology was introduced by Dolega et al. (2021). The classification takes into account the structure of the retail occupancy (presence of different subcategories of stores), vacancy rates and crime. The classification yields five groups and 15 subgroups. The geographical boundaries, as well as the typology, have been derived from data made available from the Local Data Company (LDC:http://www.localdatacompany.com/).

**Figure 2.**
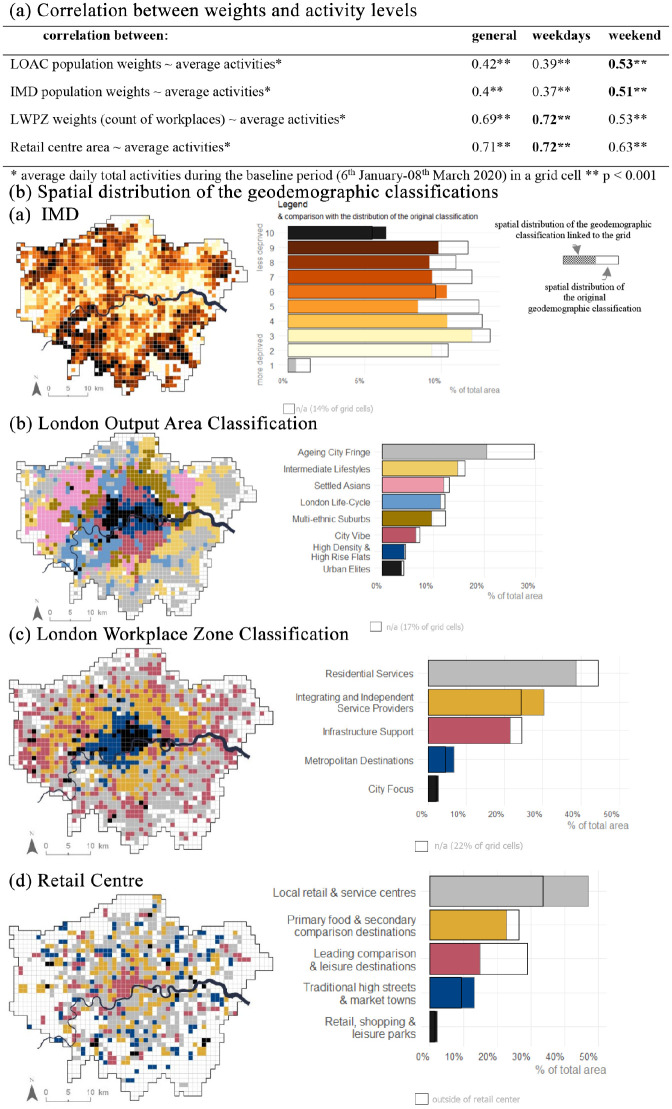
Results of linking geodemographic classification to aggregated mobile locations data. (a) The positive correlation between retail centre areas and average activities shows that the activity levels are higher in the larger retail centres. Similarly, the activity levels are higher in the grid cells with more workplaces. (b) Bar charts indicate the assignment of cells to classifications, those unfilled suggest a slight under-estimate of the number of areas in that category, whilst bars extending beyond the black borders indicate where more cells than expected were assigned to a category.

The distinct geographical units of each of the chosen classifications meant that direct linkage to 1 km^2^ grid cells used for aggregating activity data was not possible. Therefore, a simple overlay approach was taken utilising population-weighted centroids to weight the allocation of each classification category to each grid cell. We do this by overlaying the grid with population-weighted centroids that serve as a reference point for the centre of the population in an OA/WPZ. Population-weighted centroids have been calculated by the ONS and can be downloaded from their geoportal (https://geoportal.statistics.gov.uk/). Centroids are first joined with the most recent population statistics, which for Output Areas are midyear population estimates for 2018 (ONS, 2019) and for Workplace Zones are the count of workplaces in 2015 (ONS, 2016). After overlaying centroids with the 1 km^2^ grid, we perform point in polygon operation to match each geodemographic zone to the grid cell that contains its centroid. Next, we calculate the weights expressed as the total population or number of workplaces for each geodemographic classification category in each grid cell. Finally, the highest weighted category of every geodemographic classification in each grid cell is assigned as a classification type to the grid cell. There is a small number of grid cells that do not overlap with population-weighted centroids. Those areas are located mainly in the suburban areas where population density is lower and where OA/WPZ cover larger areas. In those cases, a geodemographic classification is not assigned to the grid cells (marked as n/a in Figure 2). We acknowledge that this approach might be further improved by a more sophisticated fuzzy matching methodology and the apportionment of multiple categories to each grid cell, but we felt that this was beyond the scope of our largely exploratory analysis.

Unlike the geodemographic classifications, the retail centre typology is not a population-based metric. Instead, it represents distinctive areas of increased concentration of retail activity. Therefore, the areal overlap between retail centres and grid cells is more important. To link the retail centre typology to the gridded data, we calculate the geographic coverage of the retail centre in every grid cell, aggregate the results based on the typology and assign the typology which covers the largest area as a variable for the grid cell.

To evaluate the population/area-weighted methods for assigning classification type to the grid cells, we calculate the correlation between the weights and the average daily activity levels in the pre-lockdown period (6 January to 8 March 2020). We find a significant positive correlation between the weights and the activity levels. The weights that represent the daytime population, such as the count of workplaces, yield a stronger correlation than the weights that represent residential population, such as population count used to assign IMD Deciles and LOAC. These findings comply with our previous observations that show the in-app activity data used in this case study are similar to the transit station, workplace and other activity metrics that represent ambient population. The correlation results are shown in Figure 2a.

The results of linking the geodemographic classifications to the grid cells are evaluated by comparing the areal distribution of the classification groups assigned to grid cells against the areal distribution of the classification when the original area unit (Workplace Zone or Output Area) is used (see Figure 2b). The biggest discrepancies are present at the Retail Centre Typology, where the coverage of the *Local Retail & Service Centres* group has been overestimated by ∼15% compared to the original distribution and the distribution of the *Leading Comparison & Leisure Destinations* group has been underestimated by approximately 15%. All in all, linking the geodemographic classification to 1 km^2^ grid cells using population-weighted centroids yields good results.

Across all the classifications, there are in total 28 geodemographic and related variables linked to the 1 km^2^ grid cells. Each grid cell can have a maximum of four geodemographic variables (one from each classification shown in Table 1). Geodemographic variables are now joined with the aggregated activity data based on the grid cell ID which is the common denominator between the two datasets.

#### Rescaling and aggregating data

**Figure 3.**
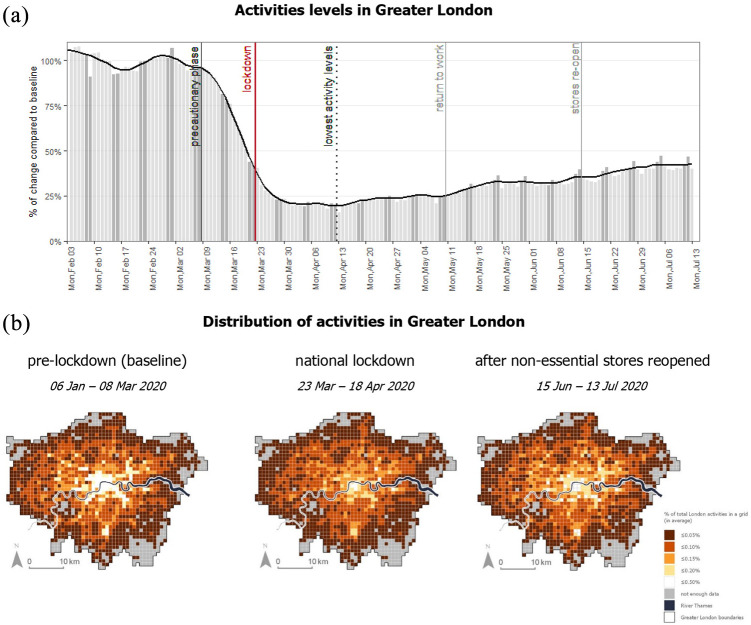
Subset of the activity data used in the case study. (a) Temporal trends during the study period. (b) Spatial distribution of the activities. The maps show the activities in a grid cell as a percentage of total activities in Greater London. The percentages are calculated for each day and then averaged across the period.

The daily total activities in each grid cell are rescaled using the methodology proposed in the Google Mobility reports, where each weekday is compared against the median value of the same weekday during the baseline. We use a baseline period of 6 January to 8 March (pre-lockdown period, see Figure 3). In essence, rescaling converts the data into percentages that show the ratio between activity levels at a given date and the baseline activity levels.

#### Segmented regression model

We run a segmented regression analysis to evaluate disparities between geodemographic classification types. This estimates intervention effects in interrupted time series studies (Wagner et al., 2002) and is often used in health research (Taljaard et al., 2014). We split the study period into five segments: 1) Baseline period before COVID-19 (5 January to 8 March), 2) Precautionary behaviour before lockdown (9–22 March), 3) National lockdown (23 March to 9 May), 4) Easing of lockdown measures phase 1 (10 May to 14 June) and 5) Easing of lockdown measures phase 2 (14 June to 13 July).

Geodemographic variables are compared against the average activity levels (= reference level) in Greater London (Figure 3). Significant and positive estimates indicate that the activity levels at this geodemographic classification type declined less compared to the reference level, meaning those areas remained more active relative to the London average. The regression is run separately for each classification to avoid multi-collinearity between the variables.

### Results

#### Exploratory analysis

The pre-lockdown period (6 January to 8 March) can be characterised by busy workdays and quiet weekends. The activity levels started to significantly decline in week 10 (9–15 March), and by the time the lockdown was announced on Monday 23 March the activity levels were already down by 56% compared to Monday 9 March. The steep decline in activities slowed down after one week of nationwide lockdown (around 27 March). The decline continued at a slower pace until reaching the lowest levels during week 16 (13–19 April), when activities were down by over 84% compared to pre-lockdown activity levels. The activity levels started to slowly recover in mid-April – weeks before any of the restrictions were officially eased. The activities recovered to 47% of pre-lockdown levels by the beginning of July.

The maps in Figure 3b show the distribution of activities during the baseline period (6 January to 8 March), when central London and the transport hubs (e.g. Croydon in south London) were the major activity hotspots; during the national lockdown (23 March to 18 April), when the distribution of activities was more equal across Greater London but some of the transport hubs remained busy (e.g. Stratford); and finally during the period after non-essential stores reopened (14 June to 13 July), when central London become the hotspot for activities again, although not at the same magnitude as in the pre-lockdown period.

#### Segmented regression analyses findings

There were no significant deviations from the average activity levels in any of the geodemographic classification groups during the baseline period. Starting from 9 March and before any mobility restrictions had been put in place, the activities in the *City Focus* (−13.60%), *Metropolitan Destinations* (−9.54%) and *Leading Comparison & Leisure Destinations* (−6.37%) declined significantly more compared to the Greater London average. These categories also sustained a steeper decline once the national lockdown was announced on 23 March. Such declines would be expected since they are characterised as having few residents and many more mobile groups such as tourists, workers and shoppers.

During the national lockdown, the activities decline more than the London average in affluent residential neighbourhoods in central London labelled *Urban Elites* (−20.11%) and in affluent suburbs labelled *London Life-Cycle* (−11.52%). On the other hand, there was significantly less decline in activity levels in struggling suburban areas classed as *Intermediate Lifestyle* (11.53%) and *Multi-Ethic Suburbs* (9.72%). Further areas that remained busy during the lockdown were the *Integrating and Independent Service Providers* (4.96%) and *Residential Services* (2.32%) type of workplace zones, as those types of jobs need to be carried out on-site.

IMD Deciles showed a clear trend – more deprived areas (IMD Decile 2 and 3) remained busier than the average during the lockdown, whereas less deprived areas (IMD Decile 8–10) had more decline in activities. Furthermore, the recovery of activities once the restrictions were eased was faster in the higher deciles and slower in the lower deciles.

The recovery was also slower in *Primary Food & Secondary Comparison Destinations* (from 1.78% during lockdown to −0.25% after reopening of non-essential retail) than in *Leading Comparison & Leisure Destinations* (−15.23% to −10.11%), and slow in *City Focus* workplace zones (−32.16% to −29.08%). Amongst the residential neighbourhoods, the recovery was fastest in *Urban Elites* (−20.11% to −13.28%).

**Figure 4.**
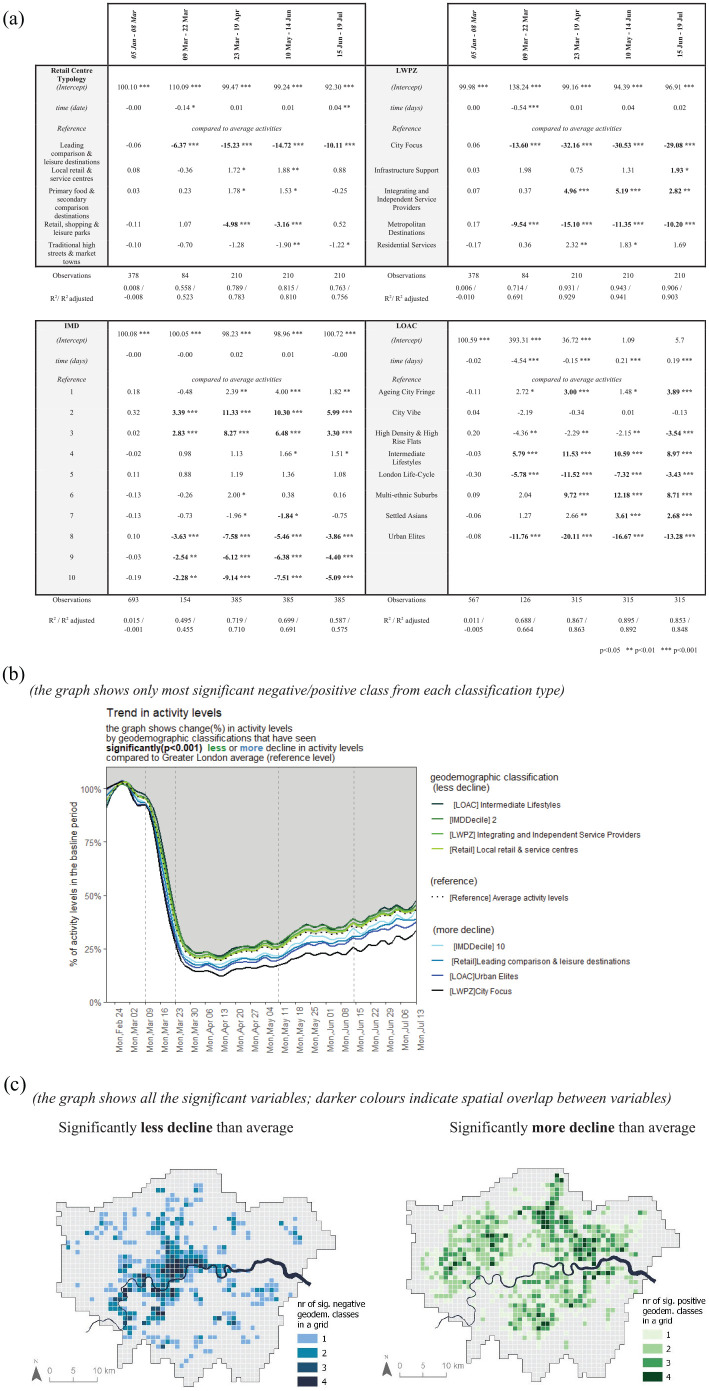
Regression results. (a) Adjusted *R*^2^ value shows the percentage of variation in the change of activity levels during this time period that the model is able to explain. The model performs poorly for the baseline period but well for the lockdown period. (b) The trendlines of the geodemographic variables that had a significant effect on the activity levels in the lockdown period. (c) Spatial distribution of the significantly positive and significantly negative estimates. Areas where the activities declined more than the average are clustered to the centre of the city whereas suburban areas remained relatively busier.

Figure 4c shows the spatial distribution of the geodemographic classification groups where the decline in activity levels remained significantly lower or higher compared to the Greater London average once the first restrictions were eased in May 2020. The map shows that the geodemographic areas that remained less busy are located in central London (e.g. Mayfair) and in affluent neighbourhoods in south London (e.g. Wimbledon). This was probably because of the lack of tourist population (especially in central London) and due to the fact that people living in those areas continued working from home. The areas where the decline in activity levels was lower than the Greater London average are located in struggling neighbourhoods in north-east, north-west and south London. This is probably because the population in those areas had to travel to work once the restrictions had been eased.

#### Limitations

Although we remain positive about using mobile location data in public health research, some limitations need to be considered when interpreting the results. First, we assume unbiased distribution across age groups, although it is likely that the age distribution is skewed towards the younger population who are more likely to own a smartphone and/or be active users of apps. People without smartphones tend to already be marginalised, so making public policy based on mobile location data can further exacerbate this. Next, we count activities by all the devices and do not distinguish between tourists and residents because our data are aggregated in a way that no individuals can be detected or tracked, making extracting any devices based on their previous behaviour not possible.

Also, there are well-studied drawbacks to geodemographic classifications – notably the ‘ecological fallacy’– that will mean only group characteristics can be assigned to individuals. Furthermore, our current regression model does not account for spatial autocorrelation between the neighbourhoods. This can be improved by using a spatial analysis technique such as geographically weighed regression.

## Conclusions

This article offers an early example of utilising location data captured through mobile applications to study short-term changes in population activity dynamics during the COVID-19 lockdown. In a wider public health context, it demonstrates how such data could support situational awareness across prolonged time periods at a granular spatial scale. By linking the mobile location data to the broader demographic characteristics, we were able to provide additional insights into the impacts of mobility restrictions in different demographic groups across Greater London. It is hoped that our analysis can offer a more nuanced insight into why the effectiveness of social distancing interventions appeared to vary between areas. The data also signals those areas likely to require the most support during a post-pandemic recovery phase as activity is slower to return.

Our analysis reveals the division between areas dominated by white- and blue-collar jobs, the latter showing a much smaller reduction in activity during the lockdown. This highlights a divide between those in jobs that can be done from home and those with jobs that must be carried out on-site, with activity levels suggesting that those working in financial services, in particular, are in a better position to work remotely. This will have important implications for transport planning and retailers, as staggered working hours mixed with homeworking where possible have drastically reduced demand in certain neighbourhoods. Comparison between different types of retail centres shows less reduction in activity levels at the *Local Retail & Service Centres*, whereas the activity levels dropped as much as 70% in the *Leading Comparison & Leisure Destinations* located commonly in dynamic central locations. The findings suggest that recovery and the challenges faced by traditional high streets and leisure centres will be very different, and local trading patterns may depend upon behaviours developed during the lockdown period (e.g. more people will be working from home) as well as upon further regulations (e.g. local lockdowns, travel restrictions).

Our analysis was finalised in September 2020 using data until July 2020; however, the data are being collected continuously so the analysis could be extended to reflect later developments, such as the further lockdowns and locally targeted interventions. Furthermore, as good-quality COVID-19 testing and mortality data are now being published, the activity levels could be inspected in relation to the positive cases and mortality rate or excess deaths in the neighbourhoods.
